# Genome-wide scans for selective sweeps using convolutional neural networks

**DOI:** 10.1093/bioinformatics/btad265

**Published:** 2023-06-30

**Authors:** Hanqing Zhao, Matthijs Souilljee, Pavlos Pavlidis, Nikolaos Alachiotis

**Affiliations:** Faculty of EEMCS, University of Twente, Enschede, The Netherlands; Faculty of EEMCS, University of Twente, Enschede, The Netherlands; Institute of Computer Science, Foundation for Research and Technology-Hellas, Heraklion, Greece; Faculty of EEMCS, University of Twente, Enschede, The Netherlands

## Abstract

**Motivation:**

Recent methods for selective sweep detection cast the problem as a classification task and use summary statistics as features to capture region characteristics that are indicative of a selective sweep, thereby being sensitive to confounding factors. Furthermore, they are not designed to perform whole-genome scans or to estimate the extent of the genomic region that was affected by positive selection; both are required for identifying candidate genes and the time and strength of selection.

**Results:**

We present ASDEC (https://github.com/pephco/ASDEC), a neural-network-based framework that can scan whole genomes for selective sweeps. ASDEC achieves similar classification performance to other convolutional neural network-based classifiers that rely on summary statistics, but it is trained 10× faster and classifies genomic regions 5× faster by inferring region characteristics from the raw sequence data directly. Deploying ASDEC for genomic scans achieved up to 15.2× higher sensitivity, 19.4× higher success rates, and 4× higher detection accuracy than state-of-the-art methods. We used ASDEC to scan human chromosome 1 of the Yoruba population (1000Genomes project), identifying nine known candidate genes.

## 1 Introduction

Positive selection plays a critical role in shaping the evolution of species. The identification of genes affected by positive selection can shed light on the forces that drive adaptation (Ohta 1996) and has important practical applications, such as identifying drug-resistant mutations in pathogens (De Groot and Bontrop 2013) and designing more effective drug treatments (Alam et al. 2011). In the presence of positive selection, an allele is favored by natural selection and its prevalence increases in a population until it is fixed. Due to genetic hitchhiking (Smith and Haigh 1974), the frequency of nearby neutral alleles linked to the selected locus also increases, creating a region with reduced variation. Because genetic diversity in the neighborhood of the favored allele is swept away by selection, this process is commonly referred to as a “selective sweep”.

Detecting traces of positive selection that has acted in the past in a population relies on finding distinct signatures left in the genomes by a selective sweep. These genetic signatures, according to the classic selective sweep model described by Smith and Haigh (1974), are (i**)** a shift in the site frequency spectrum (SFS) toward low- and high-frequency derived variants (Braverman et al. 1995; Fay and Wu 2000), (ii**)** a distinct pattern of linkage disequilibrium (LD) where high LD is found on each side of the selection target and low LD is found between loci that are located on different sides of the selection target (Kim and Nielsen 2004), and (iii**)** reduced genetic diversity in the region surrounding the selected locus. Several methods can be used for selective sweep detection, ranging from compute-inexpensive summary statistics, such as Tajima’s D (Tajima 1989) and Fay and Wu’s H (Fay and Wu 2000), to more advanced, likelihood-based approaches, such as SweepFinder2 (DeGiorgio et al. 2016) and SweeD (Pavlidis et al. 2013). They serve as neutrality tests because their distribution in the presence of selection differs from the expected distribution under neutrality.

Early neutrality tests were designed to only detect one of the aforementioned selective sweep signatures. For instance, Fay and Wu’s H (Fay and Wu 2000) detects regions with a large number of high-frequency derived variants using outgroup information to distinguish between low- and high-frequency derived variants. SweepFinder2 (DeGiorgio et al. 2016) implements a composite likelihood ratio test (Nielsen et al. 2005) that detects the deviation of the SFS in a selective sweep region from the expected SFS under the standard neutral model (Kimura 1971). Ideally, each neutrality test would only be sensitive to the sweep signature it was designed to detect. In practice, however, neutrality tests are frequently confounded by various factors such as demographic changes in population size or migration between adjacent populations (Weigand and Leese 2018), which generate spurious data patterns that resemble those expected to have been introduced in the genome by a selective sweep. Rapid population growth following a severe population bottleneck (sharp reduction in size), for instance, reduces genetic variation and the occurrence of intermediate-frequency alleles while increasing low-frequency alleles, thereby resembling two of the classic sweep signatures. Confounding factors represent a major challenge in studies that aim to provide evidence of positive selection and detect the selection target.

More recently, studies started applying a variety of alternative techniques to yield more robust analyses in the presence of confounding factors. Pavlidis et al. (2013) and Vasilarou et al. (2021) combined the results of several neutrality tests under the rationale that “the more results agreeing on an outcome, the more likely the outcome” to identify common-outlier, selective sweep regions in the first human chromosome and the SARS-CoV-2 virus genome, respectively. Alachiotis and Pavlidis (2018) proposed a composite evaluation method that relies on single-nucleotide polymorphism (SNP) vectors (polymorphic columns of a multiple sequence alignment) and considers all three classic selective sweep signatures in the evaluation of genomic regions. Pybus et al. (2015) and Schrider and Kern (2016) employed supervised machine learning using a predefined set of summary statistics as input variables to learn to identify data patterns that can be used to distinguish between neutrality and selection. The aforementioned techniques attempt to exploit the aggregate power of multiple tests for pattern recognition with the aim to increase sensitivity when searching for selective sweeps, while remaining robust to confounding factors. Yet, these approaches can be sub-optimal because the independently obtained results of different neutrality tests may be correlated, to a certain extent, if they depend on the same underlying coalescent tree (Kim and Nielsen 2004).

The most recent approaches in selective sweep detection perform pattern recognition using convolutional neural networks (CNNs) (LeCun et al. 1998; Krizhevsky et al. 2012), a class of deep neural networks that have proven highly effective and are already widely deployed in various fields, such as image, video, speech, and audio recognition (LeCun et al. 2015). CNNs process labeled multidimensional data arrays, e.g. 1D arrays for sequences or 2D arrays for images, to extract and learn to identify meaningful features (training). Thereafter, the learned knowledge of a trained CNN can be applied to produce quantitative predictions for data arrays that are previously unknown to the CNN (inference). In population genomics, CNN-based frameworks leverage information from summary statistics (1D arrays) or from aligned sequence data represented as images (2D arrays). Kern and Schrider (2018) presented diploS/HIC, a CNN-based method for classifying genomic windows into neutral regions, hard sweeps, or soft sweeps using a multidimensional vector of summary statistics (represented as an image) calculated from the window to be classified. Chan et al. (2018) developed a method for processing raw population genomic data to accurately localize recombination hotspots, i.e. genomic regions with increased recombination rate (Petes 2001). Similarly, Torada et al. (2019) developed a classification pipeline to detect and quantify natural selection from raw population genomic data. An exploratory study with a broader scope by Flagel et al. (2019) assessed the effectiveness of CNNs for various problems in population genomics: the detection of introgression (gene flow between species), the estimation of recombination rates, the detection and categorization of positive selection, and the inference of demographic information about a species’ population size history. The authors concluded that CNNs frequently match or outperform current methods in terms of accuracy.

The aforementioned studies that deploy CNNs for positive selection (Kern and Schrider 2018; Flagel et al. 2019; Torada et al. 2019) cast the detection problem as a classification task for a limited number of genomic regions, i.e. they do not thoroughly scan the entire dataset to accurately localize the selection target or estimate the extent of the genomic segment that has been affected by positive selection. Although it is possible to use these approaches to scan whole genomes, additional programming effort is required because they are not designed to perform whole-genome scans; diploS/HIC, for instance, evaluates 11 genomic windows and does not offer users the flexibility to modify this number. Furthermore, the aforementioned studies do not facilitate the discovery and deployment of new CNN designs that can potentially yield more accurate scans for selective sweeps given the data at hand. Efforts to facilitate the development of neural networks for population genetic data and the implementation of complex simulation models have only been reported very recently, but the availability of such frameworks is currently very limited (Adrion et al. 2020; Sanchez et al. 2023).

To this end, we developed a bioinformatics pipeline with a CNN at its core, dubbed ASDEC (Accurate Sweep Detection Enabled by a CNN), that can be used to build custom CNN models for genomic-region classification and selective sweep detection, and easily deploy them to scan whole genomes for traces of positive selection. Using simulations for a wide range of non-equilibrium evolutionary models, we performed a hyper-parameter optimization search to find a custom CNN architecture that is suitable for population genetic data. We used ASDEC for region classification, observing comparable classification accuracy with another CNN-based framework designed for region classification [diploS/HIC (Kern and Schrider 2018)], but an order of magnitude shorter processing times; the performance advantage of ASDEC comes from the direct use of raw sequence data instead of relying on summary statistics (diploS/HIC calculates 12 summary statistics). ASDEC is able to perform genomic scans for hard selective sweeps more accurately and with higher sensitivity than state-of-the-art selective sweep detection methods. Examining confounding factors that present major challenges to existing tools (Alachiotis et al. 2012; Pavlidis et al. 2013; DeGiorgio et al. 2016; Alachiotis and Pavlidis 2018), we observed that ASDEC is more robust to population bottlenecks, migration, and recombination hotspots. To showcase ASDEC, we scanned the first human chromosome of the Yoruba population [1000Genomes dataset (Sudmant et al. 2015)] and identified a number of candidate genes (top 0.5%) for which we report what has been discovered in the literature.

## 2 Methods

### 2.1 Framework overview

**Figure 1.**
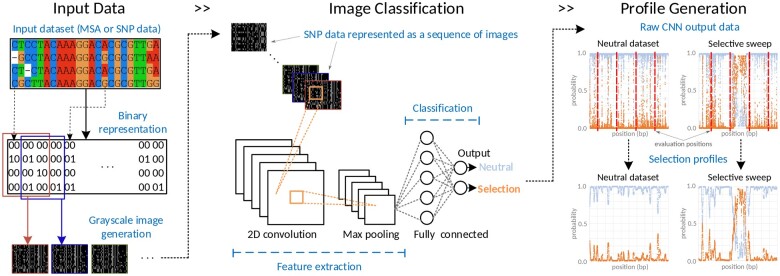
Overview of ASDEC for CNN-based selective sweep detection using a pre-trained CNN model. A multiple sequence alignment or SNP data are converted to grayscale images (Input Data) that are classified by a CNN (Image Classification) by assigning each image a probability of belonging to the “Neutral” class and a probability of belonging to the “Selection” class. The array of “Selection”-class probabilities is used in a post-processing step to generate a “Selection” profile (Profile Generation). (a) Evolutionary models included in ASDEC that contain a selective sweep and a population bottleneck with varying severity and duration. (b) Evolutionary models included in ASDEC that simulate a sweep under migration (61–70, increasing population join time) or recombination (92–96, increasing recombination intensity).

ASDEC is a processing pipeline implemented in Python. It uses Keras (Chollet et al. 2015), a high-level API to build and explore machine learning models, and the TensorFlow 2 (Abadi et al. 2016) library as the back end for training and inference of deep neural networks. ASDEC employs a CNN that consists of three combined layers (a convolutional layer and a pooling layer paired together) with the same filter size (32) and a dense layer of size 32. This CNN was developed through a multi-step hyper-parameter optimization process that involved a comprehensive exploration study that assessed different network architecture design choices, such as the number of combined layers (2, 3, 4, 5), the filter size (8, 16, 32, 64) and form (increasing/decreasing), and the number (1, 2) and size (16, 32, 64) of dense layers. Deploying a pre-trained machine learning model, ASDEC can scan whole genomes to provide estimates of the physical location and the extent of selective sweeps. Furthermore, ASDEC calculates several evaluation metrics that, in combination with the fast model-experimentation capabilities of Keras and TensorFlow 2, facilitate the search for new neural models for selective sweep detection and other population genomics problems where image classification techniques on sequence data can be exploited. A high-level overview of the framework is provided in Fig. 1.

### 2.2 Input data

Input sequence data in ms (Hudson 2002), FASTA, or VCF (Danecek et al. 2011) format are parsed, encoded into two bits per state, and stored in memory. ASDEC implements the infinite-sites model (Kimura 1969) of molecular evolution and assigns “00” to the ancestral state and “01” to the derived state, while all other states (ambiguous characters and alignment gaps) are represented by “10”. Other encoding schemes, such as one-hot-encoding, were not considered due to higher processing/memory requirements and limitations to scale with an increasing number of possible states (for instance, representing DNA and ambiguous characters would require 16 bits per state). A sliding-window algorithm with a width of *W* SNPs and a step of *S* SNPs is used to convert sequence data to a set of W×N grayscale images, where *N* is the sample size. Windows are logically placed on SNP data, thereby allowing increased granularity in SNP-dense regions while avoiding redundant operations in SNP-sparse ones. To convert a SNP window to a grayscale image, every 2-bit state (integer) is multiplied by a constant factor (127) and the result is used to form a grayscale color code. This leads to black pixels corresponding to the ancestral state (per image column), gray pixels corresponding to the derived state, and white pixels corresponding to all other states. Given *n* sequences with *T* SNPs, the sliding-window algorithm will create $L=\frac{T-W}{S}$ windows, with consecutive windows overlapping by W−S columns if S<W, or no overlap otherwise. Both the window width and the step can be determined by the user. The default width value is *W *=* *50 (empirically determined), and the window step is *S *=* *1 to create the maximum number of windows/images and achieve the maximum resolution.

Analyses of genomes that contain many missing data, e.g. sequences that have not been accurately or completely assembled/annotated, can be affected by the encoding scheme. In this case, additional investigation is needed to assess the degree to which ASDEC results may be inaccurate/misleading. A different encoding scheme to accommodate more states, e.g. differentiating alignment gaps from missing data, only requires to adapt the assignment of color codes to the encoded states accordingly. This modification will result in a more diverse color palette in the resulting images while no further changes are necessary in the rest of the ASDEC framework.

### 2.3 Image classification

In the classification stage, ASDEC can be used in one of two modes: it can either train a network architecture to generate a model, henceforth referred to as “model generation”, or it can deploy a trained CNN architecture for inference, henceforth referred to as “CNN deployment”. Notice that Figure 1 illustrates ASDEC in “CNN deployment” mode. The user friendly Keras interface is used to specify different network architectures that ASDEC will train by generating samples under various neutral/selection evolutionary models according to the standard coalescent theory (Kingman 1982; Hudson et al. 1990; Nordborg 2004). For this purpose, the framework includes an extensive list of ms (Hudson 2002), mssel (kindly provided by R. R. Hudson), msHOT (Hellenthal and Stephens 2007), and mbs (Teshima and Innan 2009) commands for various evolutionary model assumptions that have been previously used for the evaluation of selective sweep detection methods (Alachiotis and Pavlidis 2018). To perform simulations for model generation, one can either opt to employ a subset of the existing commands, or extend the command list with additional evolutionary model assumptions. Table 1 provides a summary of the evolutionary models that are included in ASDEC and can be used (referenced by dataset number) in “model generation” mode. Irrespective of the mode of operation, the output of the classification stage is a c×L numeric matrix, *R*, of posterior class probabilities, where *c* is the number of classes and *L* is the number of input data instances to the CNN. To detect positive selection in this study, we have *c *=* *2 to distinguish between a classic selective sweep (“selection” class) and a neutral genomic region (“neutral” class).

**Table 1.** The 101 evolutionary models included in ASDEC.

Ref. no.	Sweep type	Confounding factor	Software	Parameters and range [v/f]
1–60	Hard, complete	Bottleneck	ms, mssel	Severity (−*eN*): 0.005–0.5 [v]
				Duration^a^ (−*eN*): 0.0004–0.002 [v]
				Beginning^a^ (−*eN*): 0.004–0.1 [v]
				Selection coefficient (−*s*): 0.02 [f]
				Sweep start time^a^ (−*t*): 0.016 [f]
61–70	Hard, complete	Migration	ms, mssel	Population join^a^ (−*ej*): 0.003–3 [v]
				Selection coefficient (−*s*): 0.02 [f]
				Sweep start time^a^ (−*t*): 0.005[f]
71–91	No sweep	Recombination hotspot	msHOT	Hotspot region size (*v*): 5–10 kb [v]
				Hotspot intensity (−*v*): 2-100 [v]
				Mutation rate^b^ (−*t*): 2,000 [v]
				Recombination rate^b^ (−*r*): 2000 [v]
92–101	Hard, complete	Recombination hotspot	msHOT, mbs	Hotspot region size (−*v*): 5 kb [v]
				Hotspot intensity (−*v*): 2-20 [v]
				Selection coefficient (−*s*): 0.02 [f]
				Sweep start time^a^ (−*t*): 0.005 [f]
				Mutation rate^b^ (−*t*): 2,000 [v]
				Recombination rate^b^ (−*r*): 2,000 [v]

a Bottleneck duration/beginning, sweep start time, and population join time in $4N_{0}$ generations.

b Population mutation/recombination rates for the region.

v: varying parameter; f: fixed parameter.

### 2.4 Population profile

After classification, a post-processing step is applied on the probability matrix *R* to create an ASDEC population profile per class. A population profile is a 1D array of probabilities that correspond to different positions along the genome, with each entry being the average probability over a user-defined number of SNP windows that form a continuous region around the corresponding position in the genome. To generate a population profile, the user can choose among three averaging configurations: (i**)** a sliding window applied on the probability array, which allows for increased granularity in SNP-dense regions, (ii**)** a sliding window applied on genomic positions, or (iii**)** an evaluation of a series of evenly spaced genomic positions, which we henceforth refer to as grid-based evaluation. The latter facilitates the detection of common outliers between ASDEC and methods that produce grid-based score distributions along the genome [e.g. SweepFinder2 (DeGiorgio et al. 2016), SweeD (Pavlidis et al. 2013), and OmegaPlus (Alachiotis et al. 2012)].

## 3 Results

To evaluate ASDEC, we used three metrics: (i**)** sensitivity (true positive rate), (ii**)** detection accuracy (measured as the average distance between the reported sweep location and the true target), and (iii**)** the success rate (the proportion of sweeps detected within a given maximum distance from the true sweep location). A detailed description of the metrics can be found in Supplementary Section 1.

### 3.1 Classification in the presence of confounding factors

We initially evaluated the classification performance of ASDEC by comparing with the machine learning approach SURFDAWave (Mughal et al. 2020) and the CNN-based tool diploS/HIC (Kern and Schrider 2018) that are designed for classifying selective sweeps. We calculated F1-scores to measure classification performance for every evolutionary model included in ASDEC (Table 1) that contains a selective sweep and a confounding factor (population bottleneck, migration, recombination hotspot). The *F*1-score assumes values from 0.0 to 1.0, with 1.0 indicating perfect precision and recall, and 0.0 corresponding to either the precision or the recall being zero. For each of the 101 evolutionary models described in Table 1, we generated an ASDEC model using 1500 neutral simulations and 1500 simulations with a selective sweep at the center of the simulated region, as training data. We trained the model for six epochs and used the ModelCheckpoint callback of Keras to keep the model with the highest validation accuracy. The sample size was 20 sequences. For testing, we generated anew 100 neutral simulations and 100 simulations with a selective sweep at the center of the simulated region, and used the central region of each population as test data. To train diploS/HIC and ASDEC, we used 50-SNP segments from the center of the simulated regions, whereas for SURFDAWave we used segments with 650 SNPs because it cannot process populations with less than 645 SNPs. This is due to the fact that SURFDAWave needs 128 observed windows for the wavelet transformation, which requires a number of observations that is a power of two. It computes 9 summary statistics in 128 genomic windows across the region of interest, where each window consists of 10 SNPs and overlaps with its neighbors for 5 SNPs, thus requiring at least 645 SNPs. All three frameworks were trained with data that contained confounding factors, and tested using simulations with a correctly specified evolutionary model, i.e. the training and testing simulations per test had the same evolutionary model (no model misspecification). Supplementary Section 2 presents a model-misspecification analysis.

**Figure 2.**
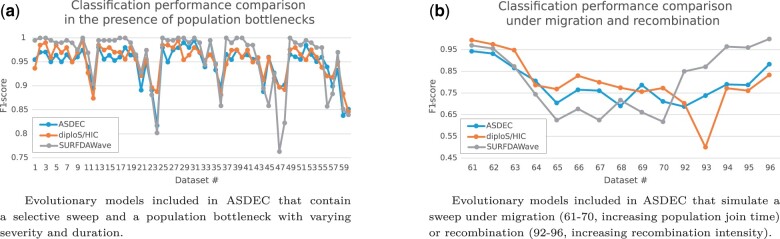
Classification performance evaluation of SURFDAWave, diploS/HIC, and ASDEC based on F1-scores.

Figure 2 shows *F*1-scores per classification method. All three ML/AI methods exhibit similar performance, with some deviations observed for some of the datasets, mainly between SURFDAWave and the other two methods. In the presence of population bottlenecks (Fig. 2a), SURFDAWave achieves slightly better performance when all tools already achieve high *F*1 scores (over 0.95), but is more sensitive to more severe bottlenecks. For a mild bottleneck (dataset 1), for instance, SURFDAWave, diploS/HIC, and ASDEC achieve *F*1-scores of 0.995, 0.936, and 0.955, respectively, whereas for a more severe bottleneck (dataset 47) diploS/HIC and ASDEC have F1 scores of 0.898 and 0.897, respectively, while SURFDAWave has an F1 score of 0.763. Classification performance deteriorates in a similar way for all methods under migration (Fig. 2b), with *F*1-scores dropping with an increasing population join time parameter (datasets 61–70). When a selective sweep occurs in a recombination region (datasets 92–96), ASDEC appears to be more robust than diploS/HIC, but all three methods are confused to a certain extent, as shown by the *F*1-scores that assume values as low as 0.619 for ASDEC (dataset 93), 0.5 for diploS/HIC (dataset 93), and 0.85 for SURFDAWave (dataset 92). SURFDAWave appears to be less sensitive to recombination hotspots than the other methods. A possible explanation for this is that SURFDAWave uses larger SNP windows and the confounding effect of recombination hotspots within such large windows is limited. Datasets 85–91 and 97–101 are not included in this analysis because they do not simulate a selective sweep.

Both diploS/HIC and ASDEC employ CNNs for classification. The former relies on the underlying selective sweep theory (Smith and Haigh 1974) by computing summary statistics to capture the evolutionary characteristics of the genomic regions that can be used for selective sweep classification, while the latter solely uses the raw sequence data and only relies on the neural network’s ability to detect and use distinctive features in the data. The fact that both approaches exhibit similar performance suggests that CNNs can potentially be used in population genetics problems with limited underlying theoretical advances. In addition, processing raw sequence data directly allows ASDEC to classify genomic regions faster than diploS/HIC since the computational cost to prepare the input data for the CNN is greatly reduced by omitting the calculation of summary statistics. Table 2 quantifies this improvement in training and inference processing times for two different evolutionary models, a mild bottleneck (dataset 1) and a more severe one (dataset 24). We used a personal off-the-shelf laptop with an Intel i7-10750H 6-core CPU at 2.6 GHz and 16 GB of main memory as a test platform (one CPU core was used). ASDEC is trained 10× faster than diploS/HIC for both datasets. Although the CNN training times are similar for both methods, computing summary statistics to train diploS/HIC is 100× slower than converting the raw sequence data to images to train ASDEC. SURFDAWave, also trained with the same data, required approximately half the time than diploS/HIC and over 5× longer than ASDEC for the mild bottleneck (dataset 1). For the more challenging severe bottleneck (dataset 24), however, SURFDAWave took considerably longer to train, becoming 3.5× slower than diploS/HIC and 34× slower than ASDEC. The inference times of all three methods do not vary noticeably with the evolutionary model. ASDEC is 5.4× faster than diploS/HIC and 3.1× slower than SURFDAWave for both datasets.

**Table 2.** Breakdown of training and inference times (in seconds) of SURFDAWave, diploS/HIC, and ASDEC for a mild bottleneck model (Dataset 1, upper table) and a severe bottleneck model (Dataset 24, lower table).

ASDEC	Training					Inference				
Dataset	Summary statistics		Image generation	CNN training	Execution time	Summary statistics		Image generation	CNN inference	Execution time
Ref. Num.: 1	Neutral class	Sweep class	(per class)			Neutral class	Sweep class	(per class)		
SURFDAWave	13.5	12.7		946.1	972.3	0.9	0.8		5.1	6.8
diploS/HIC	864.9	799.2		104.8	1768.9	58.1	54.6		1.2	113.9
ASDEC			15.5	148.2	179.2			1.1	19.1	21.3

ASDEC	Training					Inference				
Dataset	Summary statistics		Image generation	CNN training	Execution time	Summary statistics		Image generation	CNN inference	Execution time
Ref. Num.: 24	Neutral class	Sweep class	(per class)			Neutral class	Sweep class	(per class)		
SURFDAWave	13.6	12.9		6109.9	6136.4	0.9	0.8		4.9	6.6
diploS/HIC	859.9	830.8		96.5	1787.2	59.3	56.5		1.2	117.0
ASDEC			9.4	157.2	176.0			1.1	19.3	21.5

### 3.2 Detection in the presence of population bottlenecks

We evaluated the detection performance of ASDEC by comparing with the selective sweep detection tools SweeD (Pavlidis et al. 2013), SweepFinder2 (DeGiorgio et al. 2016), OmegaPlus (Alachiotis et al. 2012), and RAiSD (Alachiotis and Pavlidis 2018). For these comparisons, we used a subset of the evolutionary models included in ASDEC (due to the prohibitively long execution times of the likelihood-based tools SweepFinder2 and SweeD). For each tool, we measured the true positive rate (TPR), the success rate, and detection accuracy per evolutionary model. Each dataset comprised 100 neutral sets of SNPs and 100 sets of SNPs with a selective sweep and/or a confounding factor. The sample size was 20 sequences. ASDEC was trained for 6 epochs and 420,000 images. The other tools do not use simulated data for training; they are not ML/AI methods and, therefore, there is no training involved. Because of this fundamental difference in the underlying methods, these tools might have an advantage over ASDEC, especially when there is uncertainty about the correctness of the demographic model (with respect to the target data).

**Figure 3.**
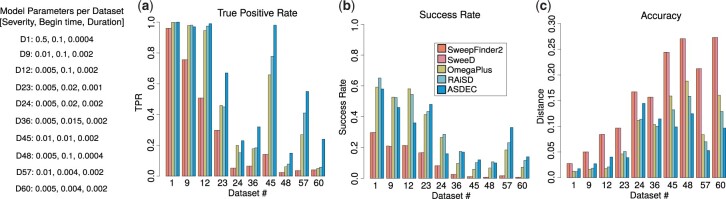
Evaluation of ASDEC based on bottleneck models with varying severity, begin time, and duration in terms of TPR for FPR = 5% (a), success rate (b), and detection accuracy (c). All success rates are based on e=1%×L. Detection accuracy is measured in terms of average distance from the selection target reported as a percentage of the genome length.

We used ms (Hudson 2002) and mssel (kindly provided by R. R. Hudson) to simulate 10 bottleneck models. Figure 3 provides the varying parameters per model (severity, begin time, duration). Each dataset comprised neutral sets of SNPs and sets of SNPs with demography and a selective sweep at the center of the simulated region. The relative population size during the bottleneck varied between 0.5 and 0.005 in comparison with the present-day population size, and the bottleneck duration ranged from 80 to 400 generations. The beginning of the bottleneck varied between 800 and 20 000 generations (backward in time), while the sweep start time was fixed at 3200 generations, including simulations where the sweep happened before, during, or after the bottleneck. Coalescent time units were converted to generations based on the assumption that the present-day population size is 50 000 diploid genomes.

Figure 3a shows a comparison of TPR for FPR = 5%. As can be observed in the figure, all tools achieve similarly high TPR in the case of recent and mild bottlenecks (datasets 1, 9, 12, and 45). More severe bottlenecks, however, present a challenge for all tools, with ASDEC exhibiting considerably higher sensitivity than the previous methods in distinguishing between neutrality and a selective sweep. For dataset 48, for instance, which simulates a severe bottleneck (severity = 0.005), the SFS-based tools SweepFinder2 and SweeD achieved TPR = 2.40%, whereas ASDEC achieved TPR = 15.0% (6.25× higher). The greatest TPR improvement over the SFS-based tools was observed for dataset 57, with SweepFinder2 and SweeD achieving TPR = 3.60%, while ASDEC achieved TPR = 55.0% (15.2× higher). For the LD-based OmegaPlus and the multi-signature-based RAiSD, dataset 60 was the most challenging evolutionary scenario, with TPR values as low as 5.10% and 5.80%, respectively. For this dataset, ASDEC achieved TPR = 24.0% (4.7× higher than OmegaPlus and 4.1× higher than RAiSD). Overall, it was observed that ASDEC exhibits similarly high TPR values with the current state-of-the-art tools for mild bottlenecks, and considerably outperforms them when more severe bottlenecks are present.

Figure 3b and C provide an evaluation of success rates and detection accuracy, respectively. Similarly to our previous observations based on TPR, ASDEC outperforms the SFS-based tools for all datasets, achieving considerably higher success rates (up to 19.4×, observed for dataset 57) while locating the selective sweep closer to its true location (up to 4× higher accuracy, also observed for dataset 57). ASDEC achieves comparable success rates with OmegaPlus and RAiSD, and higher detection accuracy for the most challenging bottlenecks (datasets 45, 48, 57, and 60). For dataset 45, OmegaPlus and RAiSD achieved their lowest success rates, 5.90% and 10.20%, respectively, while ASDEC achieved 12.0% (2× and 1.18× higher than OmegaPlus and RAiSD, respectively). The largest distance from the true selection target (lowest accuracy) of OmegaPlus and RAiSD was 18.82% and 15.85% of the genome length, observed for dataset 48. For this dataset, ASDEC located the selective sweep at distance 12.46% from the selection target, on average (33.8% and 21.4% improvement over OmegaPlus and RAiSD, respectively).

### 3.3 Sweep detection in models with migration

**Figure 4.**
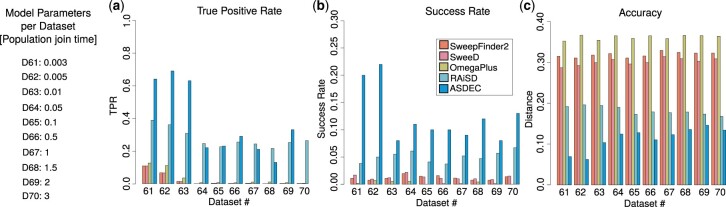
Evaluation using migration models with varying population join time: TPR for FPR = 5% (a), success rate (b), detection accuracy (c).

We used ms (Hudson 2002) and mssel to simulate a continent-island model for the migration models. Figure 4 provides a summary of the varying parameter per migration model (population join time). The continent has an effective population size that is 20× larger than the effective population size of the island, and acts as a ghost population, i.e. only sequences from the island are available. The migration rate $M_{\text{ic}}=4N_{c}m_{\text{ic}}$, where $N_{c}$ is the continent’s effective population size and $m_{\text{ic}}$ is the fraction of the island population that consists of immigrants from the continent, was set to 3. The two populations merged at time $t_{m}$ (measured in $4N_{c}$ generations), which varied between 0.003 and 3 in our simulations, thereby implementing population divergence models that ranged from very recent ($t_{m}=0.003$) to very old ones ($t_{m}=3$). The population mutation rate $\theta =4N_{0}\mu$ and population recombination rate $\rho =4N_{0}r$ were set to 2000 for the entire simulated region.

Figure 4a shows a comparison of TPR rates for FPR = 5%. As can be observed in the figure, the SFS-based methods are highly confounded by migration, with the highest TPR being as low as 10.80%, observed for the most recent divergence model (dataset 61, $t_{m}=0.003$). For the same dataset, OmegaPlus and RAiSD achieved TPR of 12.60% and 38.78%, respectively, while ASDEC achieved TPR = 64.0% (5.9×, 5.1×, and 1.65× higher than SweepFinder2/SweeD, OmegaPlus, and RAiSD, respectively). For older divergence models, the tools that rely on only one sweep signature (SweepFinder2 and SweeD rely on the SFS, and OmegaPlus relies on LD) perform poorly, while ASDEC exhibits similar TPR performance with RAiSD that relies on three signatures.

Figure 4b and c provides an evaluation of success rates and detection accuracy, respectively. It can be observed that ASDEC outperforms all other tools for all datasets, both in terms of success rate and detection accuracy. For the most recent divergence model, ASDEC achieves a success rate that is 5.26× higher than the previously highest success rate, which was achieved by RAiSD (20.0% versus 3.80%). ASDEC also achieves 1.94× higher success rate than RAiSD for the oldest divergence model. In addition, ASDEC reports the sweep location closer to the true selection target than all other tools for recent divergence models, while exhibiting similar performance with RAiSD for the older ones. However, all tools appear to be confounded by migration.

### 3.4 Detection in the presence of recombination hotspots

Varying recombination rates along the genome affect the extent of LD, which can confound selective sweep detection. Increased recombination intensity in a region might create neighboring subregions with lower LD between them relative to the LD within them. This can affect FPR since ASDEC might capture consecutive regions in a recombination hotspot that have high LD within each of them but low LD between them. This is a typical selective sweep signature and, expectedly, will resemble the selective-sweep training data. To investigate this possibly confounding effect, we used msHOT (Hellenthal and Stephens 2007) to generate neutral simulated datasets with and without recombination hotspots, and mbs (Teshima and Innan 2009) to simulate recombination hotspot models with a selective sweep. We simulated neutral evolutionary models with a single 5-kb or 10-kb recombination hotspot, and a model with three 5-kb recombination hotspots, with recombination intensity ranging from 2 to 100 (relative to the rest of the genome). Furthermore, we simulated evolutionary models with a 5-kb recombination hotspot in the middle of a 100-kb simulated region (recombination intensity ranging from 2 to 10 relative to the rest of the genome), and a selective sweep either at location 50 kb (in the recombination hotspot) or at location 30 kb (outside the recombination hotspot). ASDEC was trained using data from neutral and selective-sweep regions that were outside recombination hotspots.

To assess the effect of recombination hotspots on the FPR (false positive rate), we set cut-off values based on neutral models without recombination hotspots (95th percentile) and examined the FPR using the neutral models with recombination hotspots. We found that recombination hotspots have negligible effect on the FPR, as shown in Table 3 (the expected FPR is 5%). Neither ASDEC nor the signature-based tools are considerably misled into identifying a recombination hotspot as a selective sweep, but ASDEC is slightly more sensitive than the other methods, on average (average FPR for SweepFinder2: 3.81%, SweeD: 3.78%, OmegaPlus: 5.84%, RAiSD: 4.93%, ASDEC: 6.44%). In general, recombination hotspots do not affect the FPR. However, there were two instances where the FPR for ASDEC doubled. These occurred in regions with very high recombination intensity (10 and 100 times higher than the rest of the genome) and a small hotspot region (5 kb).

**Table 3.** The effect of recombination hotspots on FPR.

Dataset no.	71	74	77	78	81	84	85	88	91
Recombination region size	5 kb			10 kb			5 kb × 3		
Recombination intensity	2	10	100	2	10	100	2	10	100
SweepFinder2	3.9	4.4	3.8	3.8	3.6	4.2	3.7	3.6	3.3
SweeD	3.9	4.4	3.7	3.7	3.5	4.2	3.7	3.6	3.3
OmegaPlus	5.9	5.4	7.0	5.1	6.2	5.9	5.6	5.4	6.1
RAiSD	5.1	5.5	5.6	6.4	4.0	5.2	4.9	3.1	4.6
ASDEC	6.0	11.0	10.0	7.0	4.0	5.0	3.0	4.0	8.0

**Figure 5.**
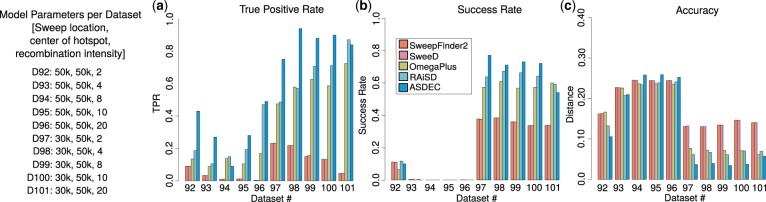
Evaluation based on models with varying recombination intensity and a selective sweep inside the recombination region (D92–D96) and outside the recombination region (D97–D101): TPR for FPR = 5% (a), success rate (b), detection accuracy (c).

To assess the power of ASDEC to locate a selective sweep in the presence of a recombination hotspot, we measured TPR for FPR = 5% (Fig. 5a), the success rate (Fig. 5b), and detection accuracy (Fig. 5c). Irrespective of the intensity of recombination in the hotspot, neither ASDEC nor any of the signature-based tools can distinguish between selection and neutrality when the selective sweep is simulated in the recombination hotspot region (datasets 92–96 in Fig. 5). When the selective sweep is outside the recombination hotspot (datasets 97–101), all methods achieve higher TPR and success rates, and lower distance error. Figure 5a shows that, for the SFS-based tools (SweepFinder2 and SweeD), TPR reduces with increasing recombination intensity in the recombination hotspot while success rates and accuracy remain more or less constant. The rest of the tools exhibit similar behavior, with TPR, success rate, and accuracy being less affected by the intensity of recombination in the recombination hotspot. For dataset 97 (lowest recombination intensity in our tests), the SFS-based tools SweepFinder2 and SweeD achieved TPR of 23.2% and 23.3%, respectively, success rates of 37.6% and 37.7%, respectively, and accuracy of 13.18% and 13.06%, respectively. The LD-based OmegaPlus and the multi-signature-based RAiSD achieved TPR of 47.50% and 48.60%, respectively, success rates of 57.3% and 63.7%, respectively, and accuracy of 7.67% and 6.14%, respectively. ASDEC outperformed all methods, achieving a TPR of 75%, a success rate of 77%, and accuracy of 3.67%. For dataset 101 (highest recombination intensity in our tests), SweepFinder2 and SweeD achieved a TPR of 4.6% (both), a success rate of 33.8% (both), and accuracy of 14.05% and 14.01%, respectively. OmegaPlus and RAiSD achieved TPR of 72.40% and 87.10%, respectively, success rates of 59.9% and 59.1%, respectively, and accuracy of 6.11% and 6.96%, respectively. ASDEC showed comparable performance with RAiSD and OmegaPlus, with a TPR of 84%, a success rate of 54%, and accuracy of 5.75%.

### 3.5 Scan of human chromosome 1

We analysed chromosome 1 of the human genome [Yoruba population, 1000Genomes dataset (Sudmant et al. 2015)] using ASDEC to demonstrate its capacity to handle real data. Monomorphic sites (≈83% of the data) and sites that violated the infinite-sites model (≈4.2% of the data) were discarded. The default window width (*W *=* *50) and step (*S *=* *1) were used for data preparation, and the “selection” profile was generated using the grid-based configuration, evaluating 800 000 positions along the chromosome (10 samples) by averaging 5000-bp genomic regions. Table 4 provides a list of nine candidate genes with high scores (top 0.5%) that have been previously identified as targets of positive selection using other methods: iHS (Voight et al. 2006), dN/dS (Kryazhimskiy and Plotkin 2008), nSL (Ferrer-Admetlla et al. 2014), CMS (Grossman et al. 2010), and GRoSS (Refoyo-Martínez et al. 2019). Another seven candidate regions were discarded (not identified by other studies). Supplementary Section 3 shows the results for the whole chromosome.

**Table 4.** Nine candidate genes in human chromosome 1 with top 0.5% ASDEC scores (Yoruba, 1000Genomes, GRCh37/hg19 assembly).

Gene name	Position (start-end, in bp)	Region identified by ASDEC	Previous candidate gene identification and method used
SCMH1	41,492,871-41,627,104	41,570,580-41,625,444	Johnson and Voight (2018), iHS
VAV3	108,113,782-108,507,545	108,244,669-108,652,426	Scheinfeldt et al. (2012), iHS
MAGI3	113,933,475-114,224,924	114,013,341-114,284,515	Ji et al. (2020), iHS
SPAG17	118,496,288-118,727,848	118,716,729-118,742,182	Kuhlwilm and Boeckx (2019), n/a
FCRL2	157,715,523-157,746,922	157,570,369-157,926,03	Matos et al. (2021), dN/dS
ALDH9A1	165,631,449-165,667,900	165,085,221-165,724,772	Landini et al. (2021), nSL
DISP1	222,988,431-223,179,337	223,016,450-223,038,632	Wagner (2007), dN/dS
CDC42BPA	227,177,566-227,505,826	227,177,271-227,199,703	Grossman et al. (2010), CMS
SLC35F3	234,040,679-234,460,262	234,368,359-234,383,563	Refoyo-Martínez et al. (2019), GRoSS

## 4 Conclusion

We presented ASDEC, a standalone framework that performs genome-wide scans for positive selection with higher sensitivity, success rate, and detection accuracy than state-of-the-art methods. To the best of our knowledge, ASDEC is the first CNN-based tool that can handle whole genomes and additionally localize the selection target and estimate the extent of the selective sweep. ASDEC can serve as the building block for designing neural architecture search strategies to discover new CNN architectures for population genomics problems.

ASDEC achieves higher detection accuracy and an order of magnitude higher sensitivity and success rates than widely used tools. It delivers similar performance with state-of-the-art tools for mild population bottlenecks, and considerably outperforms them in the case of severe bottlenecks. ASDEC is more robust to migration than methods that rely on a subset of the known selective sweep signatures. It is also robust to recombination hotspots in neutral data, and has higher performance than existing tools in detecting selection when the target is not in a recombination region. When the selection target is in a recombination hotspot, however, all methods are confounded. Finally, we used ASDEC to scan the first chromosome of the Yoruba population and identified nine candidate genes that have been previously reported as selection targets.

We performed an extensive simulation study and a series of benchmark experiments. The large number of simulations under various parameters can pave the way for fostering better practices in the evaluation of approaches in population genetics (ML/AI and others). In general, ASDEC and other ML/AI methods perform better than non-ML/-AI approaches. This might be due to the fact that specific data features are extracted (and learned) from both neutral and selection data, whereas non-ML/-AI methods use neutral data only to determine a threshold. Another reason is that these methods either exploit certain data features based on the theory of selective sweeps (SweepFinder2, SweeD, OmegaPlus) or rely on the simplistic combination of multiple features (RAiSD), while summary-statistic-free ML/AI methods like ASDEC, extract distinctive features from the data without relying on theory.

## Supplementary Material

btad265_Supplementary_Data

## Data Availability

All code and data are available at the following links: https://github.com/pephco/ASDEC and https://figshare.com/articles/dataset/Datasets-for-CNN-evaluation/22434445.
