# Supplementary material for: Genome-wide scans for selective sweeps using convolutional neural networks
Source: Bioinformatics. 2023 Jun 30;39(Suppl 1):i194–203. doi: 10.1093/bioinformatics/btad265 (PMC10311404; doi:10.1093/bioinformatics/btad265)
Supplement: btad265_Supplementary_Data [file btad265_supplementary_data.pdf]

Genome-wide Scans for Selective Sweeps using Convolutional Neural Networks [Supplementary Material]

Hanqing Zhao[1], Matthijs Souilljee[1], Pavlos Pavlidis[2], and Nikolaos Alachiotis[1]

[1]**University of Twente, Enschede, The Netherlands**
[2]**Foundation for Reseach and Technology-Hellas, Heraklion, Greece**

1 EVALUATION METRICS

ASDEC calculates several qualitative metrics based on the "selection" profile to measure detection performance given a CNN model, which facilitates the design and implementation of custom Neural Architecture Search (NAS) strategies. These metrics are a) sensitivity (true positive rate), b) detection accuracy, and c) the success rate. Furthermore, ASDEC reports the total execution time and a time breakdown per task per thread (when multiple CPU cores are used for faster processing). Given N population simulations with the true selection target at position X, an indicative measure of detection accuracy is the average distance, $Dist$, between the reported position and X, which is measured using Equation 1 as follows:

$$Dist = \frac{\sum_{i=1}^{N} |s_i - X|}{N}, \tag{1}$$

where s_i is the reported position with the highest probability in the "selection" profile of population i. Note that, because detection accuracy is quantified here in terms of distance (measured in base pairs), $Dist$ is inversely proportional to accuracy, with smaller $Dist$ values indicating higher detection accuracy. Additionally, given a simulated genome size of L base pairs and a margin of error of e base pairs from the true selection target X, the success rate Suc is measured using Equation 2 as follows:

$$Suc = \frac{\sum_{i=1}^{N} [|s_i - X| < e]}{N}, \tag{2}$$

where [ ] is the Iverson bracket notation, which returns 1 if the logical proposition expressed by the statement in brackets is true, otherwise it returns 0. All success rates reported in this study are based on $e = 1\% \times L$, i.e., 1% of the region length. Sensitivity (true positive rate) is measured as the proportion of simulated populations with selection that are correctly identified as such based on a comparison with scores obtained from neutral populations. Given N neutral populations and their "selection" profiles, a probability threshold, thr, that corresponds to a user-defined false positive rate is first determined. Then, the true positive rate, TPR, is calculated using Equation 3 as follows:

$$TPR = \frac{\sum_{i=1}^{N} [p_i > thr]}{N}, \tag{3}$$

where p_i is the highest probability in the "selection" profile of the i simulated population with selection.

2 MODEL MISSPECIFICATION

In our experimental evaluation, ASDEC models were generated based on a correctly estimated demographic model. To assess the effect of (evolutionary) model misspecification on ASDEC performance, we examined how TPR values are affected when the demographic model used for training differs from the demographic model of the dataset under test. Figure 2 presents the results of this analysis in the form of a heatmap where the diagonal corresponds to the "ideal" TPR, i.e., when the demographic model is correctly inferred and used for ASDEC model generation, while off-diagonal elements show the effect of model misspecification in relation to the corresponding diagonal value. The value in $cell\ (i,j)$ is the $log10(TPR_{ii}/TPR_{ij})$, where TPR_{ij} is the TPR when demographic model i is used for the generation of the ASDEC model (training), and demographic model j is used for the evaluation of the selective sweep (inference). Note that, while TPR_{ii} was obtained using a correctly estimated demographic model to generate simulated datasets for training, inference was performed on newly generated simulated datasets for the same demographic model to ensure that the CNN does not perform predictions on data that was already used for training. The heatmap reveals that ASDEC is relatively robust to model misspecification for mild bottlenecks, while its effect intensifies as the deviation between the training model and the test model increases, mostly resulting in lower TPR values.

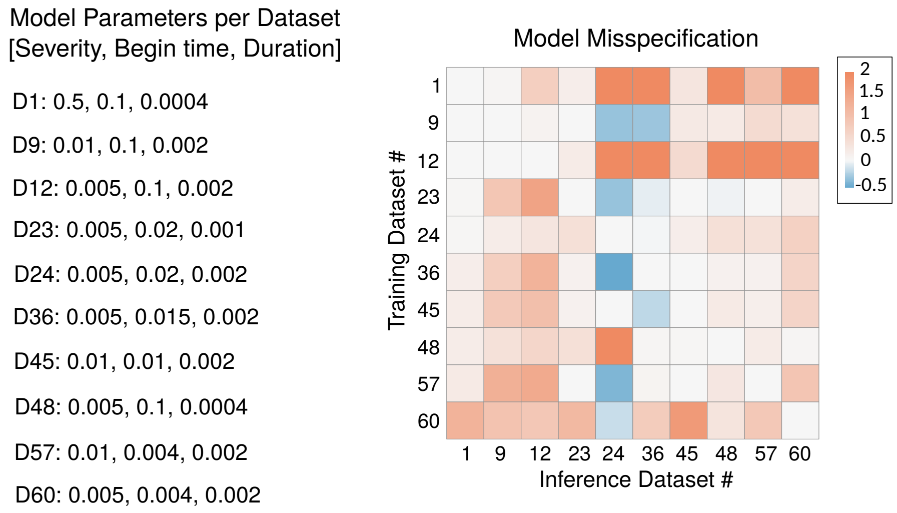

Figure 1. Evaluation of model misspecification based on bottleneck models with varying severity, begin time, and duration. The colors of the tiles represent the $log10(TPR_{ii}/TPR_{ij})$ where i is the training dataset and j is the test dataset.

3 SCAN OF HUMAN CHROMOSOME 1

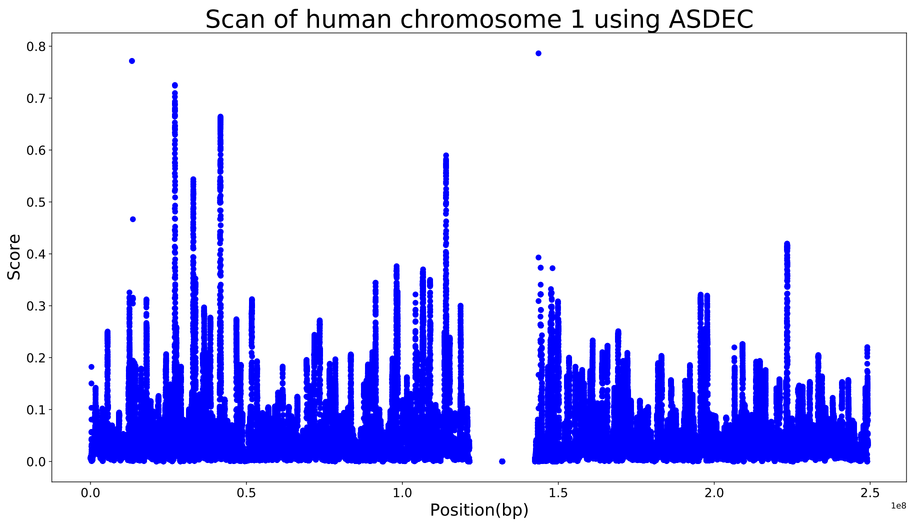

Figure 2. Scan of human chromosome 1 using ASDEC. The plot shows probability scores of the "selection" class based on grid-based post-processing of 800,000 positions along the chromosome.
